# Evaluation of Different Phenotypic and Genotypic Methods for Detection of Methicillin Resistant *Staphylococcus aureus (*MRSA)

**Published:** 2016

**Authors:** Hossein Koupahi, Sahar Honarmand Jahromy, Mohammad Rahbar

**Affiliations:** 1 *Dept. of Microbiology, Islamic Azad University, Varamin-Pishva Branch, Varamin, Iran*; 2 *Dept. of Microbiology, Iranian Reference Health Laboratory Research Center, Ministry of Health and Medical Education, Tehran, Iran*

**Keywords:** *Staphylococcus aureus*, *mecA* gene, CHOROM Agar MRSA, Oxacillin screen Agar, Methicillin Resistant

## Abstract

**Background:**

Methicillin resistant *Staphylococcus aureus* (MRSA) has been emerged as a nosocomial and community acquired pathogen worldwide. There are many challenges for laboratory detection of MRSA. The aim of this study was to compare different phenotypic methods with PCR based method as a gold standard for detection of *mecA* gene.

**Methods:**

A total of 220 clinical isolates of *S. aureus *which were isolated from various clinical specimens from September 2013 until the June of 2014 in Milad Hospital of Tehran, Iran was subject of our study. Methicillin resistance was determined by oxacillin and cefoxitin disks, oxacillin screen agar and CHROMagar™ MRSA medium. The results of these methods were compared with *mecA *gene based PCR method as a gold standard method.

**Results:**

Among 220 isolates from *S. aureus*, 105 (47.72%) isolates were positive for *mecA *gene by PCR method. The results of cefoxitin disk diffusion method with 100% sensitivity and specificity was the same as PCR method .CHROMagar™ MRSA medium had 98.13% sensitivity and 100% specificity. Oxacillin disk diffusion and oxacillin screen agar had 95.42% and 97.22% sensitivity respectively.

**Conclusion:**

Result of cefoxitin disk diffusion method with 100% sensitivity and specificity was the same as PCR method for detection *mecA* gene. Cefoxitin disk diffusion method can be used as an alternative method of PCR for detection of MRSA.

## Introduction

*Staphylococcus aureus* is an opportunistic pathogen and as a normal microbiota of the nose, skin, mouth and other parts of human body (1-2). *It* is an important agent of hospital and community acquired infections worldwide (3-5). Methicillin was first introduced in 1959 for treatment of infections caused by penicillin resistant strains of *S. aureus*. In 1961, first methicillin- resistant strain of* S. aureus* (MRSA) was reported from United Kingdom hospitals and recovered from other European countries hospitals afterwards (6-10). Infections caused by MRSA associated with a higher rate of mortality in compaction with Methicillin–susceptible *S. aureus* isolates (11). Methicillin resistance in *S. aureus* is mediated by expression of *mecA* gene, which results in production of modified penicillin–binding proteins (PBP2a).In addition recently other genes such as *femA *and other auxiliary genes are recently as the one, which can contribute to MRSA resistance (12).

In Iran, the mean prevalence of MRSA was 52.7% ±4.7 (13). For this reason, detection of MRSA in microbiology laboratories is very important in order to choose appreciates antimicrobial agents. Misidentification of MRSA leads to using of unnecessary antibiotics such as vancomycin and linezolid (14).

Appropriate and rapid identification of MRSA in clinical microbiology laboratories is essential issue for treatment and epidemiological purpose. There are many different laboratory methods for detection of MRSA (15, 16). Some of them such as detection of *mecA* gene is a gold standard method and other phenotypic methods are compared with this procedure.

The main objective of this study was to evaluate different phenotypic methods in relation to detecting *mecA* gene. This evaluation helps us for choosing a reliable routine method for detection of MRSA in our microbiology laboratories. 

## Materials and Methods

**Strains**

During September 2013 and June 2014; 220 clinical isolates of *S. aureus *were collected these strains were isolated from different clinical specimen such as blood, urine, sputum, tracheal aspirate and others. Gram stain and biochemical test such as growth on Heat-Stable nuclease, Mannitol salt agar, Coagulase, Catalase tests and susceptibility to novobiocin were used for identification of these isolates. All isolates were kept frozen at -70 °C in Trypticase soy broth containing 15% glycerol until for performance of susceptibility testing and MRSA detection.

**Detection of the** ***mecA*** **Gene by Polymerase Chain Reaction:**

Bacterial DNA was extracted by the rapid cell lysis method (18-19). First microtubes containing the bacteria in PBS were centrifuged at 10000 g for 3 min and then the supernatant was discarded. Sediment of bacteria STES and 20 ml solution 100 ml solution TE (pH = 7.6) was added to the microtube multi-tap to scale well be solved in the solution. Thirty ml lysosome were added to the mixture and were incubated at 37 °C for an hour. Then, 30 ml proteinase (K) for 30 min at 56 °C micro tubes were added. With regard to the total volume, half the volume of phenol and chloroform, add half the volume was centrifuged for 5 min at rpm 13000 rpm. After centrifugation, phase separation and the supernatant was transferred to another vial 0.1 volume of sodium acetate and 2.5 times the volume of cold absolute ethanol was added. Vials gently upside down several times, and then for 30 min in the freezer was - 20° C. Since then were centrifuged for 5 min at rpm 13000 rpm. The supernatant was discarded and 500 ml 70% ethanol was added to the vial. After a few hit again for 5 min in a centrifuge at 13,000 rpm around the supernatant was discarded and vials fixed until completely dry. In the last 100 ml was added to the solution with pH = 8 was for 18-16 h at room temperature.

The bottles after it were preserved in refrigerator. PCR – multiplex reaction for detection of *mecA* gene primers for replication were provided from Cinagene Company (Cinageneco, Tehran Iran) from this suspension, a 5 µL volume was directly used as the template for the PCR amplification of the *mecA* gene fragments.

The *mecA-F *(TCCAGATTACAACTTCACCAGG) and the *mecA*-R (CCACTTCATATCTTGTAACG) primers were used for the amplification of the 162bp fragment of the Methicillin-resistant gene (*mec A*) (11). A 50 µl PCR reaction consisted of plus 45 µl of the master mix which contained the PCR buffer (1.5X), dNTP mix (0.25mM of each), the primer (0.3 pmol), Taq DNA polymerase (0.1 U/μlit), and MgCl 2 (1.5mM) with 5 µL of the template DNA. The cycling conditions were as follows: 5 min sat 94 °C, followed by 32 cycles of denaturation at 94 °C for 50 sec, annealing at 58 °C for 50 sec, extension at 72 °C for 50 sec and the final extension step at 72 °C for 10 min.

**Fig. 1:**
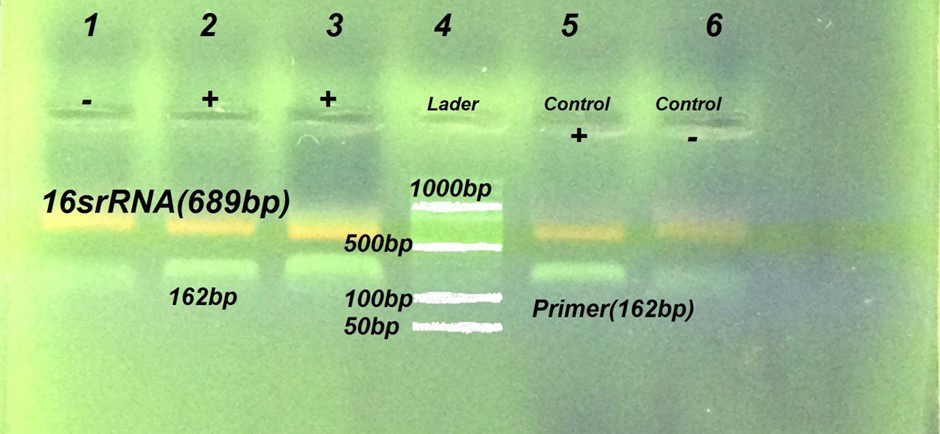
Detection of *mecA* gene by PCR method. Lane6 is negative control strain *S. aureus *ATCC: 29213, Lane 5 was positive control *S. aureus* ATCC: 33591, Lanes (1-2-3). Representative MRSA strains isolated from clinical specimens

The PCR products were visualized on a 1.5% agarose gel with ethidium bromide dye under a UV transilluminator. Amplicons of 162 bp were consistent with the *mecA* gene amplification (Fig. 1).

**Detection of MRSA by phenotypic methods**

**Disk diffusion methods**.

Disk diffusion method using oxaciliin and cefoxitin disks were performed for all isolates on Mueller–Hinton agar for detection of MRSA as recommended by CLSI guideline (20). Briefly four to five isolated colonies from overnight growth of *S. aureus *on Blood agar was suspended in n to 4-5 ml of PBS. The turbidity of suspension was adjusted to 0.5 McFarland standard turbidity and inoculated on two separate Mueller –Hinton agar plates. An oxacillin (1µg) disk aseptically placed on the Mueller–Hinton agar and incubated at 35 for 24 hours. Cefoxitin disk was placed on the other Mueller –Hinton agar and incubated at 35°C for 18 h. The inhibition zone of inhibition for each antibiotic disks were measured and by referring to CLSI guidelines reported as MRSA or methicillin susceptible *S. aureus *(20). All of antibiotic disks were provided from ROSC Company (ROSCO .Co Denmark)

**Oxacillin screen Agar**

Muller-Hinton agar plates containing 4% NaCl and 6 μg/ml of oxacillin were prepared. To perform the oxacillin screen test, a swab dipped in 0.5 McFarland’s suspension of the isolate was deposited as a spot on the agar surface and it was incubated at 35 °C for 24 h. Plates were observed carefully in transmitted light for any growth. Any growth after 24 h was considered oxacillin resistant (16, 20).

**CHROMagar™ MRSA**

**Fig. 2:**
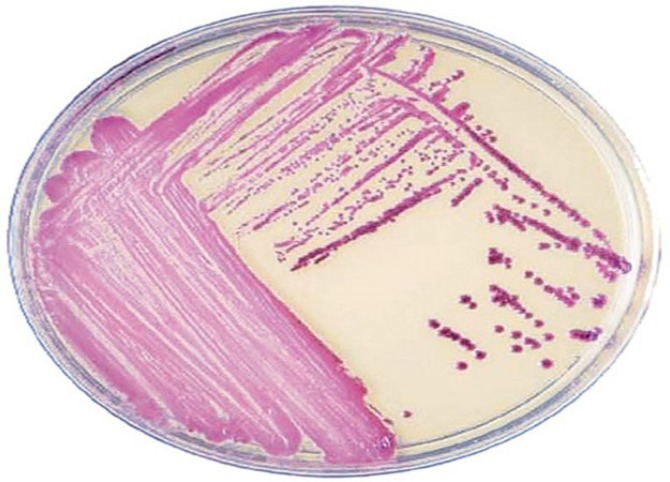
Colonies of MRSA on CHROMagar™ MRSA

 CHROMagar^TM^ MRSA for detection of MRSA was provided from CHROM agar Company, (Paris, France) The medium contained agar (15 g/liter), peptones (40 g/l), NaCl (25 g/l), and a proprietary chromogenic mix (3.5 g/l). The medium was prepared as instruction recommended by manufacturer avoiding heating at over 100 °C. Methicillin or oxacillin (4 μg/ml) was added when the agar was cooled at 48 °C. Each plate contained 20 ml of agar medium dispensed into 90-mm-diameter Petridishes. Fine isolated colony of *S. aurues* processed by direct streaking on the CHROMagar plate (Fig. 2). Plates were incubated in aerobic conditions at 37 °C for 18-24 h. Growth of intense colonies with Mauve color in 18-24 h considered as MRSA as according manufacture guideline.

**Quality control**

 The quality control strains including methicillin resistant *S aureus *( MRSA ATCC 43300 ) and methicillin sensitive *S. aureus *(MSSA ATCC 25293) were used as positive and negative controls. These strains were provided from Iranian reference health laboratory.

**Statistical analysis**

We used descriptive analysis such as percentage for categorical variables. The sensitivity, specificity, positive and negative predictive values and determining of diagnostic value of each method were calculated using Graph Pad in Stat 3.1 software.

## Results

From 74572 surveillance specimens submitted to microbiology laboratory of Milad Hospital 220 isolates *S. aureus *were recovered from clinical specimens. Out of 220 patients, 56.4% were male and 43.6% female. Among 220 *S. aureus *105 (47.72%) isolates were positive for the *mecA* gene by PCR method. Among phenotypic methods, cefoxitin disk diffusion method had the highest sensitivity and specificity. The sensitivity and specificity, of different phenotypic methods in comparison to PCR method as a gold standard procedure for detection of MRSA are shown in Table 1.

**Table1:** Comparison of different phenotypic for detection of MRSA

Method	No of MRSA	Sensitivity	Specificity
**Oxacillin Disk (1µg)**	95.45	100	100
**Cefoxitin Disk (30µg)**	100	100	105
**Oxacillin screen agar 6µg/ml**	102	97.22	100
**CHROMagar™ MRSA**	103	98.13	100
**PCR for detection ** ***mecA***	105	100	100

## Discussion

Recently increase rate of methicillin resistant strains of *S. aureus *has been posed a great difficulty in choosing suitable antibiotics for the treatment of infection caused by this microorganism (21). In Iran about 52% isolates of *S. aureus *were resistant to methicillin (13). Timely and correct identification of MRSA is very important issue for prognosis and treatment infections caused by *S. aureus *(15).There are multiple laboratory methods for detection of MRSA, however many of these methods have not sufficiently sensitivity and specificity. This may be due to the persons carrying the test and material, methods and heterogeneous resistance clinical isolates of *S. aureus *(15, 23).

Detection of *mecA* gene by molecular methods such as PCR is a gold standard method for confirmation of MRSA isolates, however, molecular detection of MRSA are not included in routine practice of many clinical microbiology laboratories (9, 24). For this reason, establishment of phenotypic methods with a high sensitivity and specificity for detection of MRSA is very essential. During last decade, there was many attempts by international guidelines such as CLSI to improve and standardization of phenotypic methods for detection of MRSA (25, 26). 

In our study, oxacillin disk diffusion method as a routine procedure for detection of MRSA revealed 100 isolatesas MRSA with 95.45% sensitivity and 100%specificity. This finding was in concordance to the results of other studies (25, 27). In spite of high sensitivity and specificity of oxacilin disk diffusion method for detection of MRSA, this method has been encountered with difficulties for detection of hetroresistant strains of *S. aureus *(26).

Other disk diffusion method in our study was using of cefoxitin disk. In present study, the cefoxitin disk diffusion method detected 105 out of 220 isolates as MRSA. Sensitivity and specificity of this method for detection of MRSA was 100%. Several studies including present study revealed that the results of the cefoxitin disk diffusion testing correlate better with the presence of *mecA* in comparison with oxacillin disk diffusion method (16 ,26,27). Cefoxitin is a better inducer of *mecA* gene expression, while oxacillin is a weak inducer of PBP2a production. Recently CLSI recommended usage of cefoxitin instead of oxacillin disk for identification of MRSA (20, 26).

Detection of MRSA by the oxacillin screen agar had 97.22% sensitivity and 100 specificity, which was concordance with other studies (25). In some cases, however heterogeneous MRSA populations of *S. aureus *are not detected on oxacillin screen agar because of their low expression of resistance. This method also is not recommended for detection coagulase-negative staphylococci by CLSI guideline (20).

Recently chromogenic media are available for detection of MRSA. (28-30). In our study, CHROMagar™ MRSA had a high sensitivity and specificity (Table 1). The results of our study were consistent with other findings for detecting of MRSA from specimens such as nasal swabs. This method is comparable by other methods such as cefoxitin disk diffusion method (31).

## Conclusion

The oxacillin disk diffusion method was less sensitive for detection of mrsa, however the results of cefoxitin disk diffusion with 100% sensitivity and specificity was the same as PCR method for detection *mecA* gene. Cefoxitin disk diffusion method can be used as an alternative method to PCR especially at those microbiology laboratories without molecular facilities.

## References

[1] Lozano C, Gómez-Sanz E, Benito D, Aspiroz C, Zarazaga M, Torres C (2011). Staphylococcus aureus nasal carriage, virulence traits, antibiotic resistance mechanisms, and genetic lineages in healthy humans in Spain, with detection of CC398 and CC97 strains. Int J Med Microbiol.

[2] Verhoeven PO, Gagnaire J, Botelho-Nevers E, Grattard F, Carricajo A, Lucht F, Pozzetto B, Berthelot P (2014). Detection and clinical relevance of Staphylococcus aureus nasal carriage: an update. Expert Rev Anti Infect Ther.

[3] Michiels B, Appelen L, Franck B, den Heijer CD, Bartholomeeusen S, Coenen S (2015 ). Staphylococcus aureus, Including Meticillin-Resistant Staphylococcus aureus, among General Practitioners and Their Patients: A Cross-Sectional Study. PLoS One.

[4] Khanal R, Sah P, Lamichhane P, Lamsal A, Upadhaya S, Pahwa VK (2015 ). Nasalcarriage of methicillin resistant Staphylococcus aureus among health care workers at a tertiary care hospital in Western Nepal. Antimicrob Resist Infect Control.

[5] Brown J, Li CS, Giordani M, Shahlaie K, Klineberg EO, Tripet-Diel JR, Ihara MS, Cohen SH (2015). Swabbing Surgical Sites Does Not Improve the Detection of Staphylococcus aureus Carriage in High-Risk Surgical Patients. Surg Infect (Larchmt).

[6] Enright MC, Robinson DA, Randle G, Feil EJ, Grundmann H, Spratt BG (2002 ). The evolutionary history of methicillin-resistant Staphylococcus aureus (MRSA). Proc Natl Acad Sci U S A.

[7] Anand KB, Agrawal P, Kumar S, Kapila K (2009 ). Comparison of cefoxitin disc diffusion test, oxacillin screen agar, and PCR for mecA gene for detection of MRSA. Indian J Med Microbiol.

[8] Strommenger B, Bartels MD, Kurt K, Layer F, Rohde SM, Boye K, Westh H, Witte W, De Lencastre H, NübelU (2014). Evolution of methicillin-resistant Staphylococcus aureustowards increasing resistance. J Antimicrob Chemother.

[9] Rahbar M, YaghoobiY, Fattahi (2006). Comparison of different laboratory methods for detection of methicillin resistant Staphylococcus naureus. Pak J Med Sci.

[10] Bhutia KO, Singh TS, Biswas S, AdhikariL (2012). Evaluation of phenotypic with genotypic methods for species identification and detection of methicillin resistant in Staphylococcusaureus. Int J Appl Basic Med Res.

[11] You JH, Ip DN, Wong CT, Ling T, Lee N, IpM (200). Meticillin-resistant Staphylococcus aureus bacteraemia in Hong Kong. JHosp Infect.

[12] Boşgelmez-Tinaz G, Ulusoy S, Aridoğan B, Coşkun-Ari F (2006). Evaluation of different methods to detect oxacillin resistance in Staphylococcus aureus and their clinical laboratory utility. Eur J ClinMicrobiol Infect Dis.

[13] Askari E, Soleymani F, Arianpoor A, Tabatabai SM, Amini A, Naderinasab M (2012). analysis. Iran J Basic Med Sci.

[14] Rodvold KA, McConeghyKW (2014 ). Methicillin-resistant Staphylococcus aureus therapy: past, present, and future. Clin Infect Dis.

[15] Velasco D, del Mar Tomas M, Cartelle M, Beceiro A, Perez A, Molina F, Moure R, Villanueva R, Bou G (2005). Evaluation of different methods for detecting methicillin (oxacillin) resistance in Staphylococcus aureus. JAntimicrob Chemother.

[16] Brown DF1, Edwards DI, Hawkey PM, Morrison D, Ridgway GL, Towner KJ, Wren MW, Joint Working Party of the British Society for Antimicrobial Chemotherapy Hospital Infection Society Association (2005). Guidelines for the laboratory diagnosis and susceptibility testing of methicillin-resistant Staphylococcus aureus (MRSA). J AntimicrobChemother.

[17] Isenberg HD (2004). Clinical microbiology procedures hand book.

[18] Unal S, Hoskins J, Flokowitsch JE, Wu CY, Preston DA, Skatrud PL (1992). Detection of Methicillin-resistant staphylococci by using the polymerase chain reaction. J Clin Microbiol.

[19] Perez-Roth E, Claverie-Martin F, Villar J, Mendez-Alvarez S (2001). Multiplex PCR for the simultaneous identification of Staphylococcus aureus and the detection of methicillin and mupirocin resistance. J Clin Microbiol.

[20] Clinical and Laboratory Standards Institute CLSI 2014 Performance standards for antimicrobial susceptibility testing; 24th informational supplement.

[21] Velasco V, Sherwood JS, Rojas-García PP, Logue CM (2014 ). Multiplex real-time PCR for detection of Staphylococcus aureus,mecA and Panton-Valentine Leukocidin (PVL) genes from selective enrichments from animals and retail meat. PLoS One.

[22] Velasco D1, del Mar Tomas M, Cartelle M, Beceiro A, Perez A, Molina F, Moure R, Villanueva R, BouG (2005). Evaluation of different methods for detecting methicillin (oxacillin) resistance in Staphylococcus aureus. J Antimicrob Chemother.

[23] Pramodhini S, Thenmozhivalli PR, Selvi R, Dillirani V, Vasumathi A, Agatha D (2011). Comparison of Various Phenotypic Methods and mecA Based PCR for the Detection of MRSA. J Clin Diag Res.

[24] Kunsang O Bhutia, T Shantikumar Singh, Shilpie Biswas, Luna Adhikari (2012). Evaluation of phenotypic with genotypic methods for species identification and detection of methicillin resistant in Staphylococcus aureus. Int J Appl Basic Med Res.

[25] Anand KB, Agrawal P, Kumar S, Kapila K (2009). Comparison of cefoxitin disc diffusion test, oxacillin screen agar, and PCR for mecA gene for detection of MRSA. Indian J Med Microbiol.

[26] Abbas Farahani, Parviz Mohajeri, Babak Gholamine, Mansour Rezaei, Hassan Abbasi (2013). 2 Comparison of Different Phenotypic and Genotypic Methods for the Detection of Methicillin-Resistant Staphylococcus Aureus. N Am J Med Sci.

[27] Rahbar M, Safadel NA (206). Evaluation of Cefoxitin Disk Diffusion Test for Routine Detection of Methicillin-resistant Staphylococcus Aureus. Iranian J Pathol.

[28] Louie L, Soares D, Meaney H, Vearncombe M, Simor AE (2006). Evaluation of a new chromogenic medium, MRSA select, for detection of methicillin-resistant Staphylococcus aureus. J Clin Microbiol.

[29] Perry JD, Davies A, Butterworth LA, Hopley AL, Nicholson A, Gould FK (2004). Development and evaluation of a chromogenicagar medium for methicillin-resistant Staphylococcusaureus. J Clin Microbiol.

[30] Han Z, Lautenbach E, Fishman N, NachamkinI (2007). Evaluation of mannitol salt agar, CHROMagar Staph aureus and CHROMagar MRSA for detection of meticillin-resistant Staphylococcus aureus from nasal swab specimen. J Med Microbiol.

[31] Ghasemian A, NajarPeerayeh S, Bakhshi B, Mirzaee M (2015 ). The Microbial Surface Components Recognizing Adhesive Matrix Molecules (MSCRAMMs) Genes among Clinical Isolates of Staphylococcus aureus from Hospitalized Children. Iran J Pathol.

